# *Bifidobacterium* compound preparations as a supplementary treatment for severe ischemic stroke: a systematic review and meta-analysis

**DOI:** 10.3389/fmicb.2025.1577898

**Published:** 2025-10-13

**Authors:** Shenghua Lu, Yunfeng Yu, Yi Liu, Huimin Zhang, Rongzhen Liu, Jianhe Liu

**Affiliations:** ^1^Department of Cardiovascular Medicine, The First Hospital of Hunan University of Chinese Medicine, Changsha, Hunan, China; ^2^Branch of National Clinical Research Center for Chinese Medicine Cardiology, The First Hospital of Hunan University of Chinese Medicine, Changsha, Hunan, China; ^3^School of Traditional Chinese Medicine, Hunan University of Chinese Medicine, Changsha, Hunan, China

**Keywords:** probiotics, *Bifidobacterium* compound preparations, *Bifidobacterium* triple viable preparations, *Bifidobacterium* quadruple viable preparations, severe ischemic stroke, meta-analysis

## Abstract

**Background:**

The benefits and risks of *Bifidobacterium* compound preparations (BCP) for patients with severe ischemic stroke (SIS) remain unclear. This study aimed to evaluate the efficacy and safety of BCP combined with enteral nutrition (EN) for SIS.

**Methods:**

Eight databases were systematically searched for relevant literature up to January 1, 2025. Two researchers independently screened the records, extracted data, and assessed the risk of bias using the Cochrane Risk of Bias Tool 1.0 (RoB 1.0). Meta-analysis, sensitivity analyses, subgroup analyses, and publication bias assessments were conducted with RevMan 5.4 software.

**Results:**

Nine randomized controlled trials and 777 patients were included in the analysis. Meta-analysis showed that regarding nutritional status, compared with the EN group, the BCP combination group significantly increased albumin (mean difference [MD] = 4.55, 95% confidence interval [CI], 3.66 to 5.45, *p <* 0.00001), total protein (MD = 7.40, 95% CI 3.64 to 11.17, *p =* 0.0001), prealbumin (MD = 46.29, 95% CI 39.60 to 52.97, *p <* 0.00001), hemoglobin (MD = 10.26, 95% CI 8.09 to 12.43, *p <* 0.00001), and transferrin (MD = 0.67, 95% CI 0.32 to 1.03, *p =* 0.0002). Regarding neurological function, the BCP combination group significantly increased the Glasgow Coma Scale score (MD = 1.86, 95% CI 1.17 to 2.56, *p <* 0.00001) and decreased the National Institutes of Health Stroke Scale score (MD = −2.17, 95% CI −3.35 to −0.99, *p =* 0.0003). Regarding intestinal barrier function, the BCP combination group significantly reduced diamine oxidase (MD = −0.69, 95% CI −0.87 to −0.50, *p <* 0.00001) and D-lactate (MD = −0.09, 95% CI −0.11 to −0.08, *p <* 0.00001). Regarding immune function, the BCP combination group significantly increased IgA (MD = 0.50, 95% CI 0.36 to 0.63, *p <* 0.00001) and IgG (MD = 3.00, 95% CI 2.03 to 3.97, *p* < 0.00001). Safety analysis revealed that the BCP combination group significantly reduced the incidence of total adverse events (risk ratio [RR] = 0.28, 95% CI 0.13 to 0.62, *p* = 0.002), pulmonary infections (RR = 0.51, 95% CI 0.33 to 0.79, *p =* 0.003), reflux (RR = 0.21, 95% CI 0.05 to 0.92, *p* = 0.04), and diarrhea (RR = 0.28, 95% CI 0.12 to 0.67, *p =* 0.005).

**Conclusion:**

BCP combined with EN can improve nutritional status, neurological function, intestinal barrier function, and immune function and reduce adverse events for patients with SIS. This approach represents a potential adjuvant treatment strategy for SIS.

**Systematic review registration:**

https://www.crd.york.ac.uk/PROSPERO/view/CRD420250653156, CRD420250653156.

## 1 Introduction

Stroke is an acute and rapidly progressing disorder caused by cerebral ischemia or hemorrhage, posing a major threat to global health (Wang et al., 2024a). According to the World Health Organization, more than 15 million people worldwide suffer from stroke annually, with ischemic stroke (IS) accounting for over 70% of all cases (Salvalaggio et al., 2023; Tuo et al., 2022). IS is characterized by its sudden onset, high disability rate, short therapeutic window, and high recurrence rate, making it one of the leading causes of disability and mortality worldwide (Patabendige et al., 2021). Severe ischemic stroke (SIS), the most critical subtype of IS, is associated with high mortality, prolonged intensive care unit (ICU) stays, and frequent complications such as infection and malnutrition (Li et al., 2023). Many patients with SIS suffer from impaired swallowing or coma, preventing adequate oral intake of food and fluids (Alsbrook et al., 2023). Consequently, enteral nutrition (EN) is typically the primary method of nutritional support for these patients. EN provides essential nutrients and energy for metabolic needs and tissue repair, while reducing the risk of aspiration-related respiratory infections (Lambell et al., 2020). However, immune suppression and intestinal barrier dysfunction in SIS often limit the effectiveness of EN, delaying neurological recovery and increasing susceptibility to infection (Ghelani et al., 2021; Zhao et al., 2022). This underscores the urgent need for adjunctive therapies that can strengthen intestinal function, improve immune regulation, and support neurological recovery.

The microbiota–gut–brain axis, a bidirectional communication network linking the gastrointestinal tract with the central nervous system, influences host physiology through neural, endocrine, and immune pathways, offering promising therapeutic avenues for neurological diseases (Feng et al., 2022; Lin et al., 2021; Raghani et al., 2024). Evidence suggests that probiotics can reduce neuroinflammation and oxidative stress by downregulating pro-inflammatory mediators and enhancing brain-derived neurotrophic factor expression, thereby facilitating neuronal repair and synaptic plasticity (Robertson et al., 2020). Among probiotic formulations, *Bifidobacterium* compound preparations (BCP), including triple and quadruple viable strains, are widely used in clinical practice. BCP have been reported to enhance intestinal barrier integrity and produce short-chain fatty acids (SCFAs), which modulate neuroendocrine signaling and may contribute to neurological protection (Indrio et al., 2024).

However, although several meta-analyses have demonstrated the beneficial effects of probiotics in stroke patients (Chen et al., 2022; Kong et al., 2024; Liu et al., 2021; Zhong et al., 2021), they have primarily evaluated probiotics as a whole, without differentiating between ischemic and hemorrhagic stroke or assessing the independent effects of specific preparations. As a result, the therapeutic value of BCP in SIS remains unclear. To address this gap, we performed a systematic review and meta-analysis to evaluate the efficacy and safety of BCP as an adjunct to EN in patients with SIS. To our knowledge, this is the first meta-analysis focusing specifically on BCP in this high-risk population. By clarifying their potential role in improving clinical outcomes, our study provides novel evidence to inform clinical practice and future guideline development.

## 2 Materials and methods

This study followed the Preferred Reporting Items for Systematic Reviews and Meta-Analyses (PRISMA) guidelines (Radua, 2021) and was registered in PROSPERO (CRD420250653156, URL: www.crd.york.ac.uk/PROSPERO/view/CRD420250653156).

### 2.1 Inclusion and exclusion criteria

Inclusion criteria: (1) Participants: Patients diagnosed with SIS. The diagnostic criteria are as follows: (i) presenting with clinical manifestations of acute cerebral infarction; (ii) accompanied by severe neurological dysfunction (NIHSS>15 points) or consciousness disorder (GCS ≤ 12 points); (iii) imaging confirming large vessel occlusion or large area of infarction; (iv) excluding hemorrhagic stroke and other non-vascular causes (Neurology et al., 2024). (2) Intervention: EN combined with BCP. BCP include *Bifidobacterium* triple viable preparations and *Bifidobacterium* quadruple viable preparations. (3) Control: EN. (4) Outcomes: Nutritional status outcomes, including albumin (ALB), total protein (TP), prealbumin (PA), hemoglobin (Hb), and transferrin (TRF), were set as primary efficacy outcomes. Included studies were required to report at least one of these nutritional outcomes. Secondary efficacy outcomes encompassed neurological functions (Glasgow Coma Scale [GCS], National Institutes of Health Stroke Scale [NIHSS]), intestinal barrier function (diamine oxidase [DAO], D-lactate [D-LA]), and immune function (IgA, IgM, IgG). Safety outcomes included adverse events such as total adverse events, pulmonary infections, intestinal infections, urinary tract infections, other infections, vomiting, reflux, refusal to eat, gastrointestinal bleeding, abdominal distension, diarrhea, and constipation. (5) Study design: Randomized controlled trials.

Exclusion criteria: (1) Duplicate studies; (2) Studies included with IS but classified as non-severe cases; (3) Studies with flawed methodological designs; (4) Studies with unavailable data.

### 2.2 Literature search

English databases including PubMed, EBSCO, the Cochrane Library, and Web of Science, as well as Chinese databases such as China National Knowledge Infrastructure (CNKI), WanFang, VIP, and SinoMed were used for literature retrieval. The search fields were set to Title/Abstract, and the search strategy was set as [(Probiotic OR *Bifidobacterium* OR Bifidobacteria OR Bacillus bifida OR Yeast OR *Saccharomyces cerevisiae* OR Saccharomyces italicus OR Saccharomyces oviformis OR S cerevisiae OR *S. cerevisiae* OR Saccharomyces uvarum var. melibiosus OR *Candida robusta* OR Saccharomyces capensis OR Lactobacillus aci4dophilus OR *Lactobacillus amylovorus* OR Lactobacill* OR lactic acid bacteria OR *Clostridium butyricum* OR Slaysophilus OR Bacillus OR Natto Bacteria OR Streptococcus thermophiles OR Enterococcus) and (Ischemic Stroke OR Ischemic Strokes OR Ischaemic Stroke OR Ischaemic Strokes OR Acute Ischemic Stroke OR Acute Ischemic Strokes OR AIS OR Brain ischemia OR middle cerebral artery occlusion OR MCA OR large vessel occlusion OR LVO OR Brain infarction OR Cerebral infarction)]. Although the study focuses on BCP in SIS, search terms for other probiotics were deliberately included to ensure comprehensiveness and avoid missing relevant studies, since some reports may not explicitly classify probiotics at the preparation level. However, during eligibility assessment, only trials using BCP met the predefined inclusion criteria and were therefore included in the final analysis. The search period covered database inception to January 1, 2025, with no language restrictions.

### 2.3 Literature screening

Two researchers (SL and YY) independently screened the records using NoteExpress (Version 3.0). Duplicates, irrelevant literature, and studies lacking complete data were excluded. Discrepancies were resolved by discussion.

### 2.4 Data extraction

Data were extracted into Excel 2010 by SL and YY independently, including study characteristics (author, year, sample size, number of males, mean age, baseline GCS, treatment type, treatment duration, and follow-up period if reported). Efficacy and safety endpoints were recorded. Extracted data were cross-checked by both investigators.

### 2.5 Risk of bias assessment

The risk of bias for each included study was independently assessed by SL and YY using the Cochrane Risk of Bias Tool 1.0 (RoB 1.0; Higgins et al., 2011). The following domains were evaluated: sequence generation, allocation concealment, blinding of participants and personnel, blinding of outcome assessment, incomplete outcome data, selective reporting, and other potential sources of bias. Each domain was judged as low, unclear, or high risk of bias. Discrepancies were resolved through discussion.

### 2.6 Statistical analysis

Meta-analysis was conducted using RevMan 5.4. For dichotomous variables, risk ratio (RR) with 95% confidence interval (CI) was calculated; for continuous variables, mean difference (MD) with 95% CI was used. Statistical heterogeneity was assessed using the I^2^ statistic, with I^2^ ≤ 50% indicating low heterogeneity and I^2^ ≥ 50% indicating substantial heterogeneity (Higgins et al., 2024). A fixed-effects model was applied when heterogeneity was low, while a random-effects model was used otherwise (Migliavaca et al., 2022). A two-sided *p* < 0.05 was considered statistically significant.

For outcomes with substantial heterogeneity (I^2^ ≥ 50%) and at least three included studies, subgroup and sensitivity analyses were conducted to explore potential sources of heterogeneity (Higgins et al., 2024). Subgroup analyses were pre-planned to assess the impact of clinical heterogeneity on specific outcomes, including factors such as the type of BCP preparation, dosage, and treatment duration. Based on the type of BCP preparation, “triple preparation” and “quadruple preparation” subgroups were established. Based on dosage, subgroups of “0.63 g ter in die (tid),” “1.5 g tid,” and “2.0 g tid” were defined. Based on treatment duration, “2 weeks” and “4 weeks” subgroups were defined. Leave-one-out sensitivity analyses were used to identify the influence of individual studies on heterogeneity and to assess the robustness of the results.

Publication bias was evaluated using Egger’s regression test in Stata 15.0 (Egger et al., 1997). Funnel plots have limited interpretability when fewer than 10 studies are available, as visual asymmetry cannot be reliably assessed. Although Egger’s test also has reduced statistical power in small samples, it offers a more objective and quantitative evaluation of potential publication bias than visual inspection alone. Accordingly, we employed Egger’s test and interpreted the results with caution.

## 3 Results

### 3.1 Literature screening results

**Figure 1:**
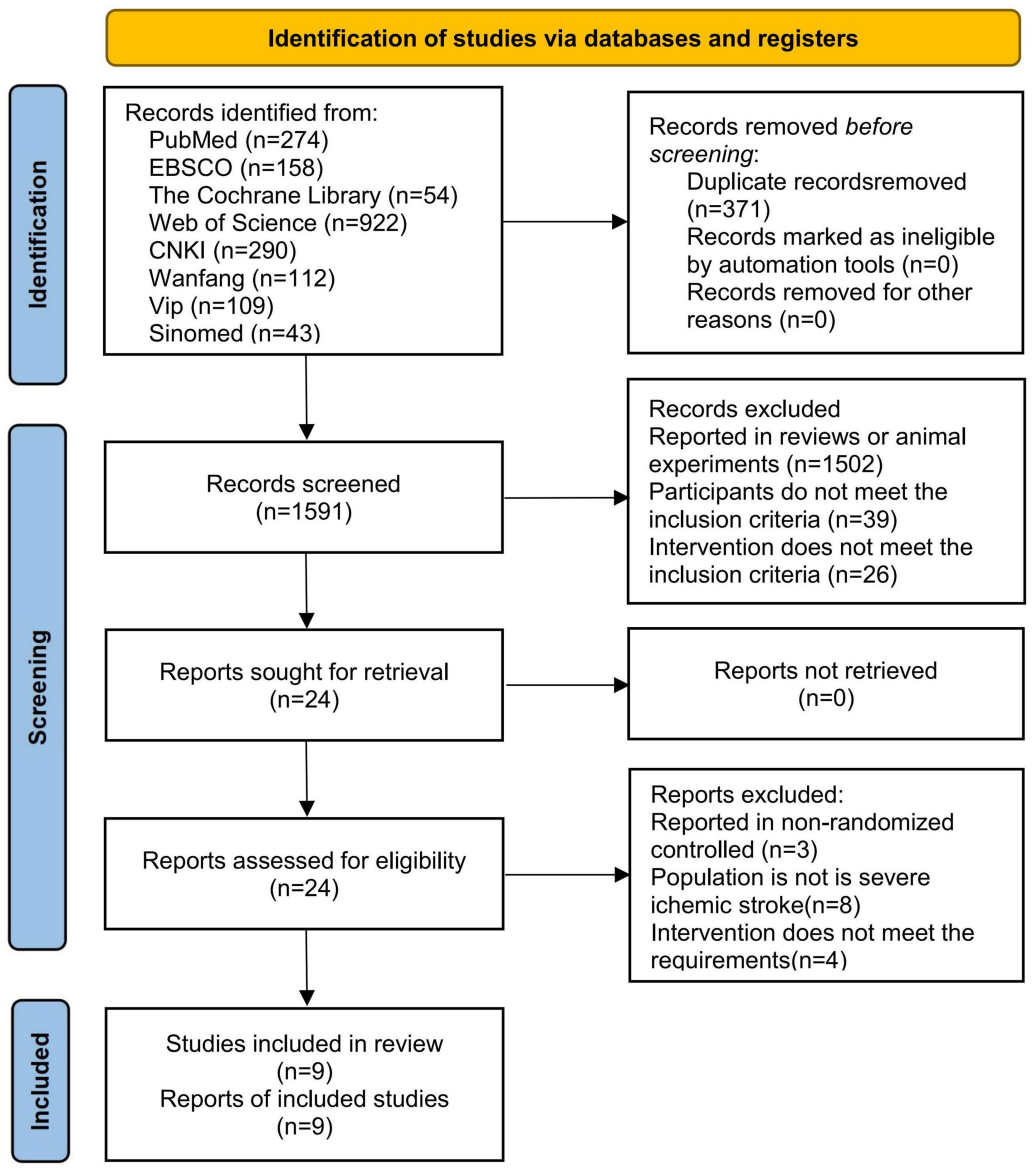
Study flow diagram.

A total of 1962 relevant articles were retrieved from the databases, including 274 from PubMed, 158 from EBSCO, 54 from the Cochrane Library, 922 from Web of Science, 290 from CNKI, 112 from Wanfang, 109 from VIP, and 43 from Sinomed. During the screening process, 371 articles were excluded due to duplication, and 1,567 articles were excluded because the topics were not relevant. Subsequently, after reviewing the full text, 15 articles were excluded for not meeting the inclusion criteria. Among them, 3 articles reported non-randomized controlled trials, 8 articles reported patients without SIS, and 4 articles reported non-conforming intervention regimens. Finally, nine studies were included (Chen et al., 2021; Dong et al., 2018; Li et al., 2021; Li and Jiang, 2024; Pang, 2016; Wan et al., 2021b; Wan et al., 2021a; Yang et al., 2018; Zhang et al., 2017). The literature screening process is shown in Figure 1.

### 3.2 Basic characteristics

Nine clinical studies and 777 patients with SIS were included in this meta-analysis. Among them, 389 people received EN, and 388 people received BCP combined with EN. All nine included studies were from China and were published between 2016 and 2024. Five studies received *Bifidobacterium* triple viable preparations combined with EN (Li et al., 2021; Pang, 2016; Wan et al., 2021b; Wan et al., 2021a; Yang et al., 2018), and 4 studies received *Bifidobacterium* quadruple viable preparations combined with EN (Chen et al., 2021; Dong et al., 2018; Li and Jiang, 2024; Zhang et al., 2017). The dosing frequency of all studies was 3 times a day, and the duration ranged from 2 to 4 weeks. The baseline data of the experimental and control groups in all included studies were comparable. The basic characteristics are shown in Table 1.

**Table 1:** Basic characteristics of included studies.

Study	Sample	Male (%)	Age (years)	GCS	Treatment	Treatment duration (weeks)	Outcomes measures
Chen et al. (2021)	50	58.0	66.5	/	Bifid quadruple viable preparation 1.5 g tid and enteral Nutrition	2	(A)(B)(C)(D)(H)(I)
	50	60.0	66.0	/	Enteral Nutrition	2	
Dong et al. (2018)	41	58.5	62.4	7.4	Bifid quadruple viable preparation 1.5 g tid and enteral Nutrition	4	(A)(B)(F)(G)(M)(N)(O)(P)(Q)(U)
	41	56.1	63.4	7.7	Enteral Nutrition	4	
Li et al. (2021)	45	46.7	68.8	8.5	Bifid triple viable preparation 0.63 g tid and enteral Nutrition	4	(A)(D)(R)(T)(U)(V)(W)(X)
	45	51.1	68.5	8.3	Enteral Nutrition	4	
Li and Jiang (2024)	60	51.7	56.1	/	Bifid quadruple viable preparation 1.5 g tid and enteral Nutrition	4	(A)(B)(C)(D)(E)(G)(H)(I)(J)(K)(L)(M)(N)(O)(P)(Q)(U)
	60	58.3	56.6	/	Enteral Nutrition	4	
Pang (2016)	48	56.3	72.1	8.9	Bifid triple viable preparation 0.63 g tid and enteral Nutrition	4	(A)(B)(F)(R)(S)(T)(U)(X)
	48	52.1	71.6	9.1	Enteral Nutrition	4	
Wan et al. (2021a)	42	57.1	57.2	/	Bifid triple viable preparation 0.63 g tid and enteral Nutrition	2	(A)(B)(D)(E)(G)(J)(K)(L)
	41	63.4	57.9	/	Enteral Nutrition	2	
Wan et al. (2021b)	28	/	/	/	Bifid triple viable preparation 2.0 g tid and enteral Nutrition	2	(A)(C)(D)(N)(R)(S)(T)(U)(V)
	30	/	/	/	Enteral Nutrition	2	
Yang et al. (2018)	30	60.0	66.2	8.3	Bifid triple viable preparation 0.63 g tid and enteral Nutrition	4	(A)(B)(D)(F)
	30	63.3	65.3	8.9	Enteral Nutrition	4	
Zhang et al. (2017)	44	54.5	/	/	Bifid quadruple viable preparation 1.5 g tid and enteral Nutrition	2	(A)(B)(C)(D)(E)(H)(I)
	44	57.3	/	/	Enteral Nutrition	2	

(A) Albumin (ALB); (B) Total protein (TP); (C) Prealbumin (PA); (D) Hemoglobin (Hb); (E) Transferrin (TRF); (F) Glasgow coma scale (GSC); (G) National institute of health stroke scale (NIHSS); (H) Diamine oxidase (DAO); (I) D-lactate (D-LA); (J) IgA; (K) IgM; (L) IgG; (M) Total adverse events; (N) Pulmonary infections; (O) Intestinal infections; (P) Urinary tract infections; (Q) Other infections; (R) Vomiting; (S) Reflux; (T) Food refusal; (U) Gastrointestinal bleeding; (V) Abdominal distension; (W) Diarrhea; (X) Constipation. tid, ter in die. The male ratio, average age, and baseline neurological function of the experimental group and the control group were comparable.

### 3.3 Risk of bias

**Figure 2:**
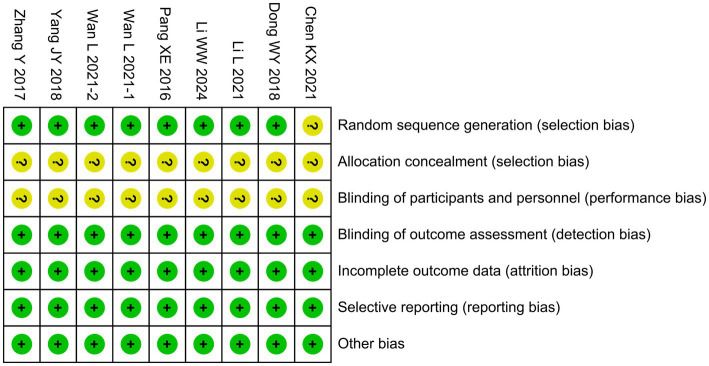
Risk of bias graph.

One trial (Chen et al., 2021) did not describe the method of random sequence generation, resulting in an unclear risk of selection bias. For allocation concealment, none of the nine studies reported how treatment assignments were concealed, raising concerns that group allocation could have been predictable. Likewise, blinding of participants and personnel was neither described nor implemented in any of the studies (Chen et al., 2021; Dong et al., 2018; Li et al., 2021; Li and Jiang, 2024; Pang, 2016; Wan et al., 2021b; Wan et al., 2021a; Yang et al., 2018; Zhang et al., 2017). This absence of blinding is particularly relevant for outcomes based on clinical assessments, such as GCS and NIHSS scores, which may have been influenced by observer expectations, thereby increasing the risk of performance bias. By contrast, the risks in other domains were judged to be low. Specifically, blinding of outcome assessment was considered low risk, as the majority of outcomes (e.g., laboratory parameters and standardized neurological scores) were objective and less likely to be influenced by the assessors’ awareness of group allocation. Incomplete outcome data were judged to be at low risk because all included studies reported outcome data for nearly all randomized participants and provided no indication of differential attrition. Selective reporting was assessed as low risk, since all prespecified outcomes described in the methods were reported in the results, and no evidence of outcome omission was identified. Finally, other potential sources of bias (e.g., baseline imbalance or funding-related bias) were not apparent. The overall risk of bias assessment is summarized in Figure 2.

### 3.4 Meta-analysis

#### 3.4.1 Nutritional status

**Figure 3:**
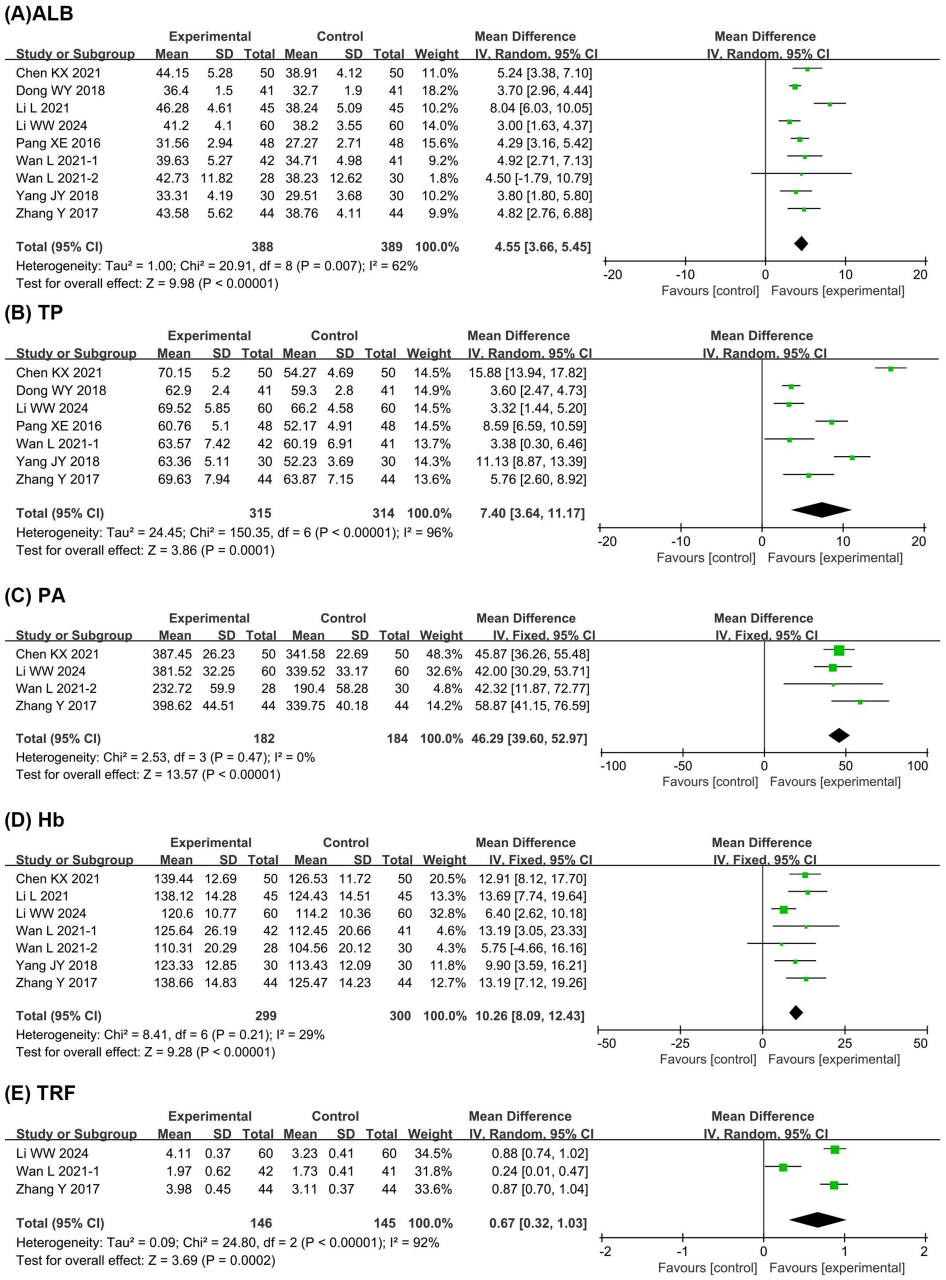
Forest plots of meta-analyses on the nutritional status. **(A)** ALB; **(B)** TP; **(C)** PA; **(D)** Hb; **(E)** TRF. ALB, albumin; TP, total protein; PA, prealbumin; Hb, hemoglobin; TRF, transferrin.

Figure 3 presents the meta-analysis results of nutritional status. It shows that compared with the EN group, the BCP combination group had significantly higher levels of ALB (MD = 4.55, 95% confidence interval [CI] 3.66 to 5.45, *p* < 0.00001, I^2^ = 62%), TP (MD = 7.40, 95% CI 3.64 to 11.17, *p* = 0.0001, I^2^ = 96%), PA (MD = 46.29, 95% CI 39.60 to 52.97, *p* < 0.00001, I^2^ = 0%), Hb (MD = 10.26, 95% CI 8.09 to 12.43, *p* < 0.00001, I^2^ = 29%), and TRF (MD = 0.67, 95% CI 0.32 to 1.03, *p* = 0.0002, I^2^ = 92%).

#### 3.4.2 Neurological function

**Figure 4:**
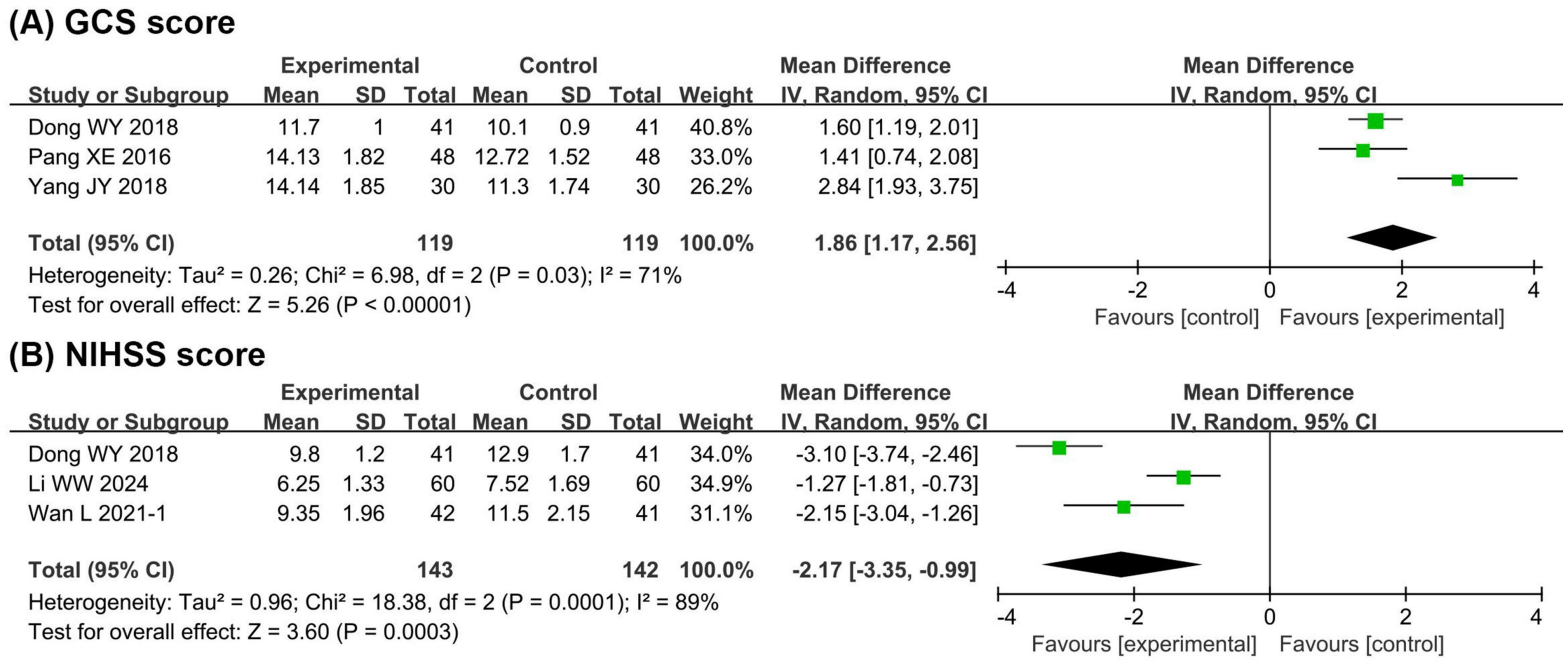
Forest plots of meta-analyses on the neurological function. **(A)** GCS score; **(B)** NIHSS score. GCS, Glasgow coma scale; NIHSS, national institute of health stroke scale.

Figure 4 presents the meta-analysis results of neurological function. It shows that compared with the EN group, the BCP combination group had significantly improved GCS score (MD = 1.86, 95% CI 1.17 to 2.56, *p* < 0.00001, I^2^ = 71%) and reduced NIHSS score (MD = −2.17, 95% CI −3.35 to −0.99, *p* = 0.0003, I^2^ = 89%).

#### 3.4.3 Intestinal barrier function

**Figure 5:**
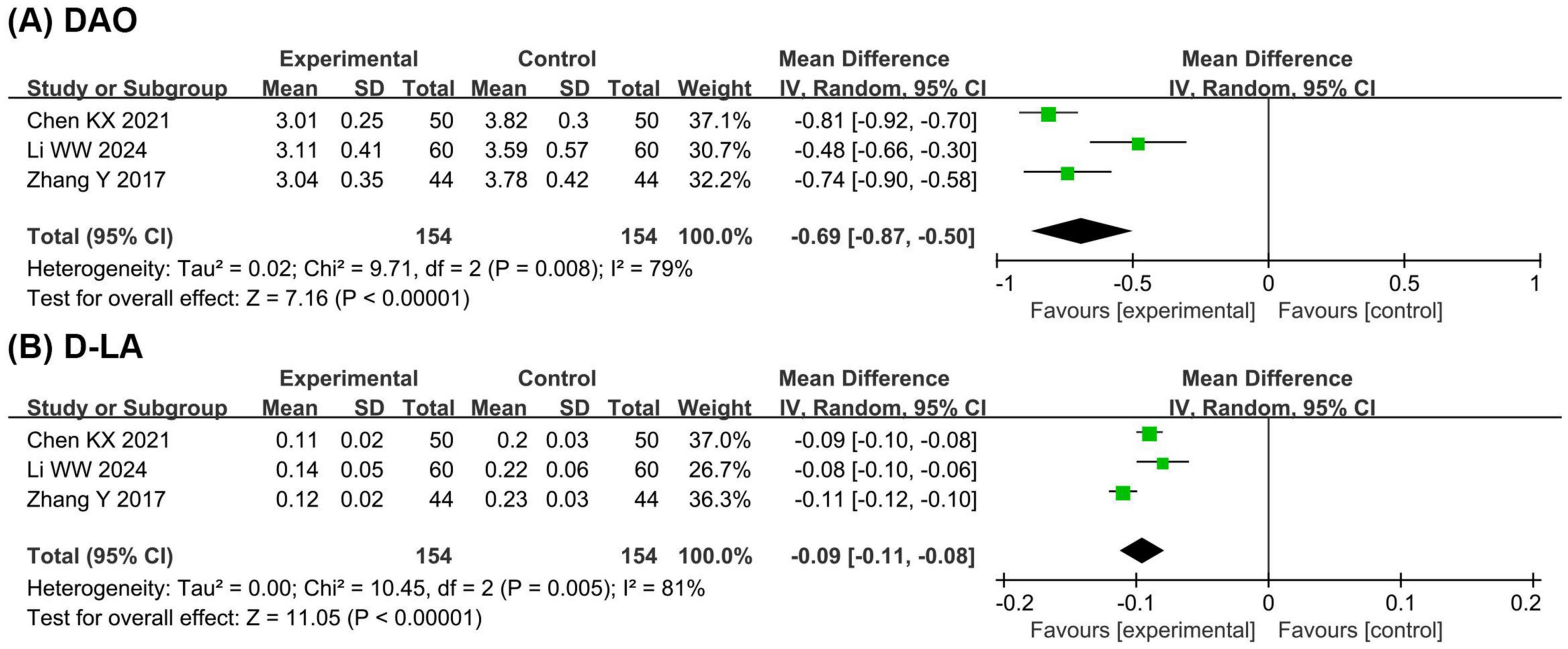
Forest plots of meta-analyses on the intestinal barrier function. **(A)** DAO; **(B)** D-LA. DAO, diamine oxidase; D-LA, D-lactate.

Figure 5 presents the meta-analysis results of intestinal barrier function. It shows that compared with the EN group, the BCP combination group had significantly lower levels of DAO (MD = −0.69, 95% CI −0.87 to −0.50, *p* < 0.00001, I^2^ = 79%) and D-LA (MD = −0.09, 95% CI −0.11 to −0.08, *p* < 0.00001, I^2^ = 81%).

#### 3.4.4 Immune function

**Figure 6:**
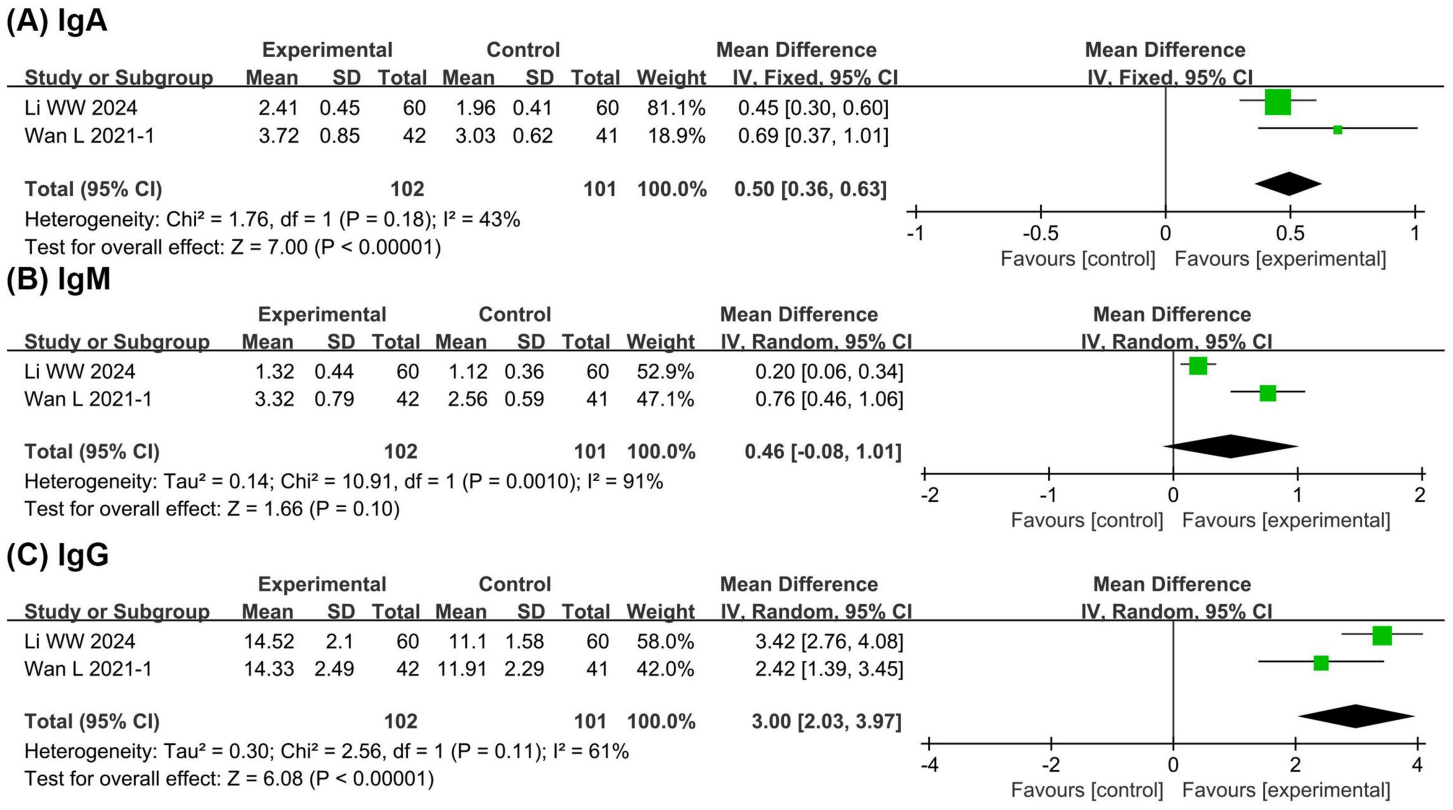
Forest plots of meta-analyses on the immune function. **(A)** IgA; **(B)** IgM; **(C)** IgG.

Figure 6 presents the meta-analysis results of immune function. It shows that compared with the EN group, the BCP combination group had significantly higher levels of IgA (MD = 0.50, 95% CI 0.36 to 0.63, *p* < 0.00001, I^2^ = 43%) and IgG (MD = 3.00, 95% CI 2.03 to 3.97, *p* < 0.00001, I^2^ = 61%) with no significant difference in IgM (MD = 0.46, 95% CI −0.08 to 1.01, *p* = 0.10, I^2^ = 91%). These findings suggest potential immunomodulatory benefits of BCP supplementation, although the evidence remains limited due to the small number of studies.

#### 3.4.5 Adverse events

Table 2 presents the meta-analysis results of adverse events. It shows that compared with the EN group, the BCP combination group had significantly lower incidence rates of total adverse events (RR = 0.28, 95% CI 0.13 to 0.62, *p* = 0.002, I^2^ = 0%), pulmonary infections (RR = 0.51, 95% CI 0.33 to 0.79, *p* = 0.003, I^2^ = 0%), reflux (RR = 0.21, 95% CI 0.05 to 0.92, *p* = 0.04, I^2^ = 0%), and diarrhea (RR = 0.28, 95% CI 0.12 to 0.67, *p* = 0.005, I^2^ = 0%). Notably, the observed reduction in pulmonary infections is of particular clinical relevance, as such infections are common complications in patients with severe ischemic stroke and can significantly affect prognosis. However, there were no significant differences in intestinal infections (RR = 0.18, 95% CI 0.03 to 0.98, *p* = 0.05, I^2^ = 0%), urinary tract infections (RR = 0.23, 95% CI 0.04 to 1.33, p = 0.10, I^2^ = 0%), other infections (RR = 0.27, 95% CI 0.05 to 1.63, *p* = 0.15, I^2^ = 0%), vomiting (RR = 0.50, 95% CI 0.22 to 1.13, *p* = 0.10, I^2^ = 0%), food refusal (RR = 0.27, 95% CI 0.05 to 1.62, *p* = 0.15, I^2^ = 0%), gastrointestinal bleeding (RR = 0.33, 95% CI 0.05 to 2.06, *p* = 0.24, I^2^ = 0%), abdominal distension (RR = 0.54, 95% CI 0.25 to 1.14, *p* = 0.11, I^2^ = 37%), and constipation (RR = 0.43, 95% CI 0.16 to 1.17, *p* = 0.10, I^2^ = 0%).

**Table 2:** Meta-analysis results for safety outcomes.

Outcome	Number of studies	Experimental (events/total)	Control (events/total)	I^2^/%	RR (95% CI)	*p* value
Total adverse events	2	7/101	25/101	0	0.28 (0.13, 0.62)	0.002
Pulmonary infections	3	16/129	33/131	0	0.51 (0.33, 0.79)	0.003
Intestinal infections	2	1/101	8/101	0	0.18 (0.03, 0.98)	0.05
Urinary tract infections	2	1/101	6/101	0	0.23 (0.04, 1.33)	0.10
Other infections	2	1/101	5/101	0	0.27 (0.05, 1.63)	0.15
Vomiting	3	7/121	15/123	0	0.50 (0.22, 1.13)	0.10
Reflux	2	2/76	10/78	0	0.21 (0.05, 0.92)	0.04
Food refusal	2	1/93	5/93	0	0.27 (0.05, 1.62)	0.15
Gastrointestinal bleeding	2	1/86	4/86	0	0.33 (0.05, 2.06)	0.24
Abdominal distension	3	8/121	16/123	37	0.54 (0.25, 1.14)	0.11
Diarrhea	2	5/73	19/75	0	0.28 (0.12, 0.67)	0.005
Constipation	3	5/121	12/123	0	0.43 (0.16, 1.17)	0.10

RR, risk ratio; CI, confidence interval.

### 3.5 Sensitivity analysis

Significant heterogeneity was observed for ALB, TP, TRF, GCS, NIHSS, DAO, and D-LA, each with at least three studies included. Therefore, sensitivity analyses were conducted to explore the potential sources of heterogeneity (Supplementary Table S1). The results showed that the heterogeneity of ALB was mainly attributable to the study by Li et al. (2021), which intervened at an earlier stage of disease (within 25 h of onset). After excluding this study, the heterogeneity of ALB markedly decreased while the result remained statistically significant (MD = 3.96, 95% CI 3.47 to 4.46, *p* ≤ 0.00001, I^2^ = 0%). The heterogeneity of TRF originated from Wan et al. (2021b), which used a *Bifidobacterium* triple viable preparation. Excluding this study substantially reduced heterogeneity while preserving statistical significance (MD = 0.88, 95% CI 0.77 to 0.98, *p* ≤ 0.00001, I^2^ = 0%). For GCS score, the heterogeneity was attributable to Yang et al. (2018), which enrolled a relatively small sample size. After its exclusion, heterogeneity decreased significantly while the effect remained robust (MD = 1.55, 95% CI 1.20 to 1.90, *p* ≤ 0.00001, I^2^ = 0%). DAO heterogeneity was linked to Li and Jiang (2024), which had a longer treatment duration (4 weeks). Excluding this study eliminated heterogeneity while maintaining statistical significance (MD = −0.79, 95% CI −0.88 to −0.70, *p* ≤ 0.00001, I^2^ = 0%). For D-LA, heterogeneity stemmed from Zhang et al. (2017), which may be explained by specific regional dietary habits of the participants. After exclusion, heterogeneity was markedly reduced and the effect remained significant (MD = −0.09, 95% CI −0.10 to −0.08, *p* ≤ 0.00001, I^2^ = 0%). However, the sources of heterogeneity for TP and NIHSS could not be identified.

Moreover, the leave-one-out sensitivity analysis showed that the results for ALB, TP, PA, Hb, NIHSS score, GCS score, D-LA, DAO, vomiting, abdominal distension, and constipation were robust, whereas the results for TRF and pulmonary infections were not robust. Specifically, after excluding the study by Zhang et al. (2017), the difference in TRF was no longer significant (MD = 0.57, 95% CI −0.06 to 1.19, *p* = 0.08). After excluding the study by Wan et al. (2021b), the difference in pulmonary infections was no longer significant (RR = 0.33, 95% CI 0.09 to 1.19, *p* = 0.09). These findings indicate that the pooled estimates for TRF and pulmonary infections should be interpreted cautiously, as they may be influenced by individual studies. However, leave-one-out sensitivity analyses were not performed for IgA, IgM, IgG, total adverse events, and several specific adverse events, because fewer than three studies were available for each. Since there was no heterogeneity in the methodological quality of the included studies, sensitivity analysis based on methodological quality was not conducted.

### 3.6 Subgroup analysis

Subgroup analyses were conducted to further investigate the sources of heterogeneity in ALB, TP, TRF, GCS, NIHSS, DAO, and D-LA. As no substantial heterogeneity was found in age or sex across studies, subgroup analyses focused on preparation type, dosage, and treatment duration (Supplementary Tables S2–S4). The results showed that TRF heterogeneity was related to preparation type and dosage. In the preparation-based subgroup analysis, both triple preparations (MD = 0.24, 95% CI 0.01 to 0.47, *p* = 0.04, I^2^ = 0%) and quadruple preparations (MD = 0.88, 95% CI 0.77 to 0.98, *p* ≤ 0.00001, I^2^ = 0%) significantly increased TRF levels. In the dosage-based analysis, both 1.5 g tid (MD = 0.88, 95% CI 0.77 to 0.98, *p* ≤ 0.00001, I^2^ = 0%) and 2.0 g tid (MD = 0.24, 95% CI 0.01 to 0.47, *p* = 0.04, I^2^ = 0%) significantly improved TRF. For DAO, heterogeneity was associated with treatment duration, as both 2-week (MD = −0.79, 95% CI −0.88 to −0.70, *p* ≤ 0.00001, I^2^ = 0%) and 4-week interventions (MD = −0.48, 95% CI −0.66 to −0.30, *p* ≤ 0.00001, I^2^ = 0%) significantly reduced DAO levels.

In summary, heterogeneity in ALB was associated with early intervention, TRF with probiotic preparation and dosage, GCS with sample size, DAO with treatment duration, and D-LA with regional dietary habits. Importantly, these sources of heterogeneity did not undermine the robustness of the overall results, supporting the reliability of the meta-analysis. Notably, no methodological or clinical sources of heterogeneity were identified for TP and NIHSS, suggesting that their heterogeneity may be statistical in origin.

### 3.7 Publication bias

**Figure 7:**
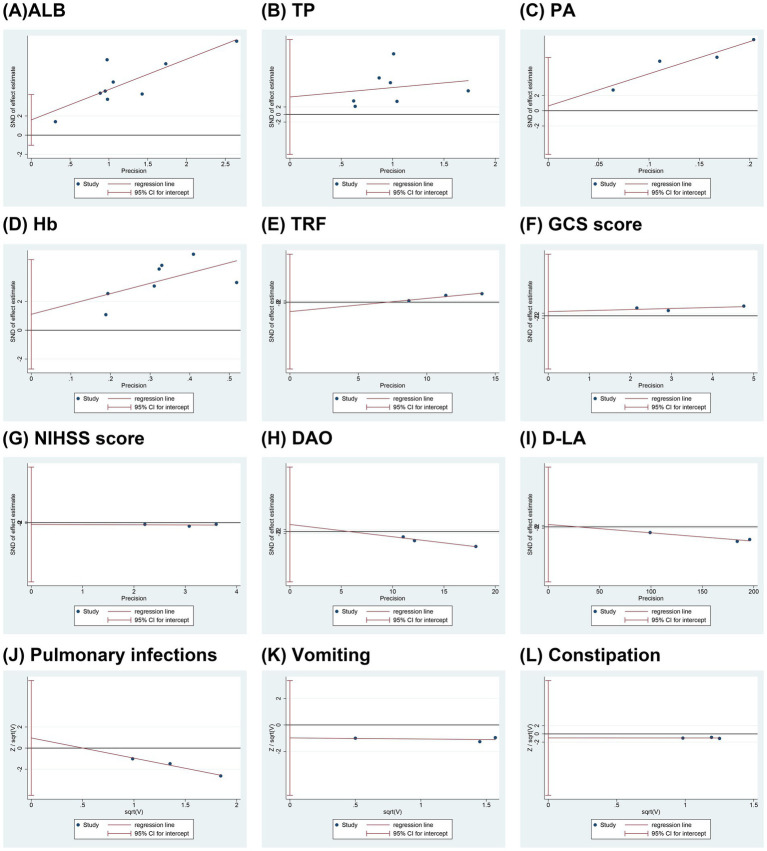
Funnel plots of publication bias. **(A)** ALB; **(B)** TP; **(C)** PA; **(D)** Hb; **(E)** TRF; **(F)** GCS score; **(G)** NIHSS score; **(H)** DAO; **(I)** D-LA; **(J)** Pulmonary infections; **(K)** Vomiting; **(L)** Constipation. ALB, albumin; TP, total protein; PA, prealbumin; Hb, hemoglobin; TRF, transferrin; GCS, Glasgow coma scale; NIHSS, national institute of health stroke scale; DAO, diamine oxidase; D-LA, D-lactate.

Egger’s tests were conducted to assess publication bias for each outcome, as illustrated in Figure 7. The results indicated no significant publication bias for the following outcomes: ALB (*p* = 0.198), TP (*p* = 0.468), PA (*p* = 0.711), Hb (*p* = 0.487), TRF (*p* = 0.284), GCS score (*p* = 0.525), NIHSS score (*p* = 0.749), DAO (*p* = 0.364), D-LA (*p* = 0.687), pulmonary infection (*p* = 0.266), vomiting (*p* = 0.230), and constipation (*p* = 0.538). However, it should be noted that each outcome included fewer than 10 studies, which may limit the statistical power and reliability of publication bias assessments. In outcomes with only two included studies, such as IgA, IgM, IgG, and certain specific adverse events, Egger’s test could not be performed, thereby further constraining the ability to identify potential publication bias. Consequently, the results of publication bias tests should be interpreted with caution, and the possibility of undetected bias cannot be excluded.

## 4 Discussion

### 4.1 Research background and significance

Immunosuppression and intestinal barrier damage are important factors affecting the supply of EN and neurological function recovery in SIS (Neurology et al., 2024). Therefore, supplementing EN with probiotic preparations represented by BCP is expected to further improve the prognosis of SIS. Although previous meta-analyses had shown that probiotics improve the prognosis of stroke (Chen et al., 2022; Kong et al., 2024; Liu et al., 2021; Zhong et al., 2021), they ignored the differences between hemorrhagic and IS. In addition, these meta-analyses treated probiotics as a whole while ignoring the characteristics of different probiotic preparations. These gaps limit the applicability of prior evidence to the clinical context of SIS. To our knowledge, this is the first meta-analysis specifically focusing on the efficacy and safety of BCP as an adjunct to EN in SIS patients. By distinguishing SIS from other stroke subtypes and by analyzing BCP as a defined therapeutic preparation rather than grouping all probiotics together, our study provides more precise and clinically relevant evidence. The findings demonstrate that BCP combined with EN could improve the nutritional status, neurological function, intestinal barrier, and immune function of patients with SIS, and reduce the incidence of total adverse events, pulmonary infections, reflux, and diarrhea.

### 4.2 Effectiveness evaluation

Regarding nutritional status, compared with the EN group, the BCP combination group significantly increased the levels of TP, ALB, PA, Hb, and TRF in patients with SIS. TP and ALB are important indicators for assessing the nutritional status of critically ill patients (Zhou et al., 2021). PA is an important indicator for early identification of protein-energy malnutrition (Pardo et al., 2023). Hb reflects the body’s protein nutritional status and anemia (Zhao et al., 2024). Therefore, the benefits of BCP in TP, ALB, PA, and Hb reflect their ability to promote the improvement of nutritional status. Furthermore, TRF is a key regulator of iron metabolism. It not only protect neurons from ischemic damage through antioxidation but also reduce further damage of iron ions to the blood–brain barrier by maintaining iron homeostasis (Hu et al., 2024; Wang et al., 2024b). Therefore, the benefit of BCP in TRF levels reflects their potential to alleviate neuronal oxidative damage. In previous meta-analyses, both Chen et al. (2022) and Zhong et al. (2021) reported that probiotics increased the levels of TP, ALB, PA, and Hb in stroke patients, and Kong et al. (2024) also pointed out that *Bifidobacterium* triple viable preparations increased TRF levels, supporting our viewpoint. The sensitivity analysis showed that the heterogeneity of ALB was mainly attributable to the study by Li et al. (2021), which implemented the intervention at an earlier stage of disease (within 25 h of onset). After excluding this study, the result for ALB remained statistically significant (MD = 3.96, 95% CI 3.47 to 4.46, *p* ≤ 0.00001, I^2^ = 0%), indicating that the finding was robust. However, in our sensitivity analysis, exclusion of the study by Zhang et al. (2017) rendered the effect of BCP on TRF non-significant (MD = 0.57, 95% CI −0.06 to 1.19, *p* = 0.08). The main distinction was that Zhang et al. (2017) enrolled patients exclusively from Sichuan Province, where dietary patterns are characterized by frequent consumption of spicy and pungent foods. Such dietary habits are known to influence gut microbiota composition and function, which in turn may affect TRF regulation. Therefore, we speculate that the regional dietary background may partly explain the sensitivity of the TRF outcome. Nevertheless, as the effect did not remain robust after sensitivity analysis, the potential benefit of BCP on TRF levels should be interpreted with caution, and further studies are needed to validate this finding. In summary, improving nutritional indicators such as TP, ALB, and Hb is crucial for enhancing recovery capacity, reducing complications, and improving overall prognosis in SIS patients.

Regarding neurological function, compared with the EN group, the BCP combination group decreased NIHSS score and increased GCS score. NIHSS score and GCS score are important tools for assessing the state of consciousness and neurological deficits in stroke patients, and their benefits confirm the role of BCP in promoting neurological function recovery. In previous meta-analyses, Chen et al. (2022) did not report the effect of probiotics on neurological function, while Liu et al. (2021) supported our findings on GCS score. The sensitivity analysis showed that the heterogeneity of GCS was attributable to Yang et al. (2018), which enrolled a relatively small sample size. After excluding this study, the effect on GCS remained statistically significant (MD = 1.55, 95% CI 1.20 to 1.90, *p* ≤ 0.00001, I^2^ = 0%), indicating that the result was robust. Additionally, Kong et al. (2024) found that *Bifidobacterium* triple viable preparations significantly improved Neuropathy Disability Score, confirming the role of BCP in promoting neurological repair from another indicator. Interestingly, Zhong et al. (2021) showed that probiotics had no significant effect on NIHSS, which was completely different from our results. This contradiction was attributed to differences in probiotic strains and stroke types. Specifically, we only included patients with SIS receiving BCP, in contrast, Zhong et al. (2021) included a wide range of probiotic species and did not limit the type of stroke. This broad inclusion criteria may introduce additional confounding factors, thus affecting the meta-analysis results of NIHSS score. Therefore, we believe that BCP are positive in improving neurological function in patients with SIS. Clinically, improvements in neurological scores translate into better functional recovery, reduced disability, and improved quality of life, which are central goals in SIS management.

Regarding intestinal barrier function, adding BCP to EN significantly reduced DAO and D-LA levels. DAO is a specific enzyme of intestinal mucosal cells, reflecting the degree of intestinal mucosal damage (Shi et al., 2025). D-LA, a metabolite of intestinal flora, indicates increased intestinal permeability (Battaglini et al., 2020). The significant decrease in DAO and D-LA levels indicates that BCP promotes the remodeling of the intestinal barrier in patients with SIS. In previous meta-analyses, Chen et al. (2022) reported the benefits of probiotics in DAO and D-LA, and Kong et al. (2024) reported the benefits of *Bifidobacterium* triple viable preparations in DAO and D-LA, consistent with our findings. Liu et al. (2021) and Zhong et al. (2021) did not report indicators related to intestinal barrier function. In addition, Kong et al. (2024) also showed that BCP significantly reduced endotoxin and endothelin levels. This further highlights the potential of BCP in restoring the intestinal barrier, although they focused on IS in a broad sense. Our subgroup and sensitivity analyses indicated that the heterogeneity of DAO was associated with treatment duration, while that of D-LA was related to dietary habits, and these clinical heterogeneities did not compromise the robustness of the results. Clinically, reinforcing intestinal barrier integrity can lower infection risk, decrease systemic inflammation, and thereby improve recovery outcomes in SIS.

Regarding immune function, our meta-analysis showed that the combination of BCP increased the levels of IgA and IgG, while having no significant effect on IgM levels, consistent with the meta-analysis results of Chen et al. (2022). However, Kong et al. (2024) found that *Bifidobacterium* triple viable preparations increased the levels of IgG and IgM in patients with IS, while having no significant effect on IgA levels. This contradictory result may be mediated by different durations. The average duration of our included studies was 3.1 weeks, while the duration of the studies included by Kong et al. (2024) was 2 weeks. In fact, IgM is the initial antibody produced by the body and plays a major role in the primary immune response; in contrast, IgA and IgG dominate the secondary immune response, taking effect more slowly but lasting longer (Li et al., 2019). Therefore, as the duration increases, the benefits of probiotics in IgM become no longer significant, while the benefits in IgA and IgG become more significant. Clinically, enhancing IgA and IgG responses may strengthen host defense, reduce susceptibility to secondary infections, and improve long-term outcomes in SIS patients.

### 4.3 Mechanism analysis

The beneficial effects of BCP observed in our meta-analysis may be explained by several interrelated mechanisms. First, BCP help preserve the intestinal barrier, consistent with our findings on reduced DAO and D-LA levels. *Bifidobacterium* promotes mucus secretion, enhances tight junction protein expression, and facilitates epithelial repair, thereby limiting bacterial translocation and maintaining gut integrity (Engevik et al., 2019; Foroni et al., 2011). Second, BCP modulate host immune responses, which aligns with the observed increases in IgA and IgG. Probiotics can balance Th1/Th2 and Treg/Th17 responses, enhance B cell function, and regulate antibody production, thus contributing to improved systemic immunity (Dargahi et al., 2019). Third, BCP exert anti-inflammatory and antioxidative effects, which may underlie their role in improving neurological recovery and TRF regulation. Short-chain fatty acids (SCFAs), key metabolites of probiotics, alleviate neuroinflammation, promote neuronal repair, and protect the blood–brain barrier through modulation of T cell polarization and inhibition of histone deacetylase activity (Fang et al., 2023). Taken together, these mechanisms provide a plausible biological explanation for our results, namely that BCP enhance nutritional status, restore intestinal barrier function, regulate immune balance, and facilitate neurological recovery in SIS patients.

### 4.4 Safety evaluation

Our meta-analysis showed that BCP combined with EN could significantly reduce total adverse events by 72% in patients with SIS. Specifically, BCP reduced pulmonary infections by 49%, while having no significant effect on intestinal infections, urinary tract infections, and other infections. Pulmonary infections are common complications and leading causes of death in SIS patients, often associated with prolonged ICU stays and increased healthcare burden (de Jonge et al., 2020). Thus, a reduction in pulmonary infections could have important clinical implications, as it may translate into improved survival rates and a decreased burden on ICU resources. However, sensitivity analysis showed that after excluding the study by Wan et al. (2021a), the difference in pulmonary infections was no longer statistically significant (RR = 0.33, 95% CI 0.09 to 1.19, *p* = 0.09). This instability indicates that the evidence should be interpreted with caution. One plausible explanation relates to treatment duration. Among the included studies, Wan et al. was the only trial with a 2-week treatment period, whereas the others used 4-week interventions. This finding suggests that the protective effect of BCP against pulmonary infections may be associated with their early antidiarrheal benefits. By reducing diarrhea in the initial treatment phase, BCP may help limit nutritional loss and thereby lower the risk of pulmonary infections. Nevertheless, given the limited number of available studies, these observations remain exploratory and require confirmation in future clinical trials.

Additionally, BCP reduced the incidence of reflux by 79% and diarrhea by 72%, while having no significant effect on vomiting, food refusal, gastrointestinal bleeding, abdominal distension, and constipation. The benefits of BCP in reflux and diarrhea may be related to its gastrointestinal barrier repair function. Interestingly, although the meta-analysis by Zhong et al. (2021) also reported the benefits of probiotics in gastrointestinal adverse events, it believed that the differences in reflux, diarrhea, abdominal distension, constipation, gastric retention, and gastrointestinal bleeding were all significant. This difference was also attributed to probiotic and stroke types, because Zhong et al. (2021) included a wide range of probiotic types and did not limit the type of stroke. Nevertheless, the existing results support that probiotics reduce the risk of pulmonary infections, reflux, and diarrhea, highlighting their value in improving the prognosis of SIS.

### 4.5 Evaluation of formulation, dose, and duration

Subgroup analysis demonstrated that in terms of preparations, both triple and quadruple preparations significantly increased ALB levels, indicating that both types of BCP could improve the prognosis of patients with SIS. In terms of dose, BCP at “0.63 g tid” and “1.5 g tid” significantly increased ALB levels, while a dose of “2 g tid” failed to achieve this benefit. However, this negative result may be mediated by a small sample size, as the “2 g tid” subgroup only included 1 study and 86 participants. In terms of duration, BCP at both “2 weeks” and “4 weeks” significantly increased ALB levels, suggesting that both short-term and medium-term treatments improved SIS prognosis. In fact, previous opinions suggested starting EN as early as possible for patients with SIS to slow the deterioration of nutritional status and reduce the 28-day mortality rate (Pardo et al., 2023; Wang et al., 2023). Therefore, we recommend starting EN combined with BCP as early as possible and continuing the treatment for 4 weeks or more.

### 4.6 Limitations and prospects

Although this study enriches the evidence for BCP in the treatment of SIS, several limitations should be acknowledged. First, the methodological quality of the included studies was inconsistent, and some studies had deficiencies in randomization, allocation concealment, and blinding, which may have introduced selection and performance biases. Second, all included trials were conducted in China, which restricts the geographical scope of the evidence. This not only limits the generalizability of the findings to other regions and populations but also raises the possibility that cultural and dietary habits, such as regional food preferences, may have influenced the observed outcomes. Third, the average age of participants ranged from 56.1 to 71.6 years, which may not fully capture the response to BCP across different age groups of SIS patients. Fourth, although this meta-analysis suggests that BCP is beneficial in SIS, the optimal dose and duration remain uncertain. Fifth, the number of included studies for each outcome was relatively small, meaning that the statistical power of Egger’s test was limited, and the assessment of publication bias may therefore be less robust.

In light of these limitations, future research should aim to: (i) further optimize study design with rigorous randomization, allocation concealment, and blinding to minimize bias and provide higher-level evidence; (ii) conduct clinical trials across diverse countries and populations to improve the external validity of the findings and clarify the influence of cultural and dietary backgrounds; (iii) explore dose–response and duration–effect relationships to determine the optimal treatment regimen for BCP in SIS; and (iv) include larger sample sizes to allow for more reliable assessments of publication bias and strengthen the robustness of the conclusions.

## 5 Conclusion

This meta-analysis demonstrates that BCP combined with EN improves nutritional status, neurological function, intestinal barrier integrity, and immune function, while reducing total adverse events in patients with SIS. The evidence is limited by several factors, including the lack of high-quality multicenter RCTs, the uncertainty regarding the optimal dose and duration of BCP, and the exclusive inclusion of Chinese populations, despite these positive findings. Future studies should employ rigorous trial designs with adequate blinding, evaluate long-term clinical outcomes, and include diverse populations to enhance generalizability and provide stronger guidance for clinical practice.

## Data Availability

The original contributions presented in the study are included in the article/Supplementary material, further inquiries can be directed to the corresponding author.
