# Supplementary material for: Bifidobacterium compound preparations as a supplementary treatment for severe ischemic stroke: a systematic review and meta-analysis
Source: Front Microbiol. 2025 Oct 13;16:1577898. doi: 10.3389/fmicb.2025.1577898 (PMC12554666; doi:10.3389/fmicb.2025.1577898)
Supplement: Supplementary file 1 [file Table_1.docx]

**Supplementary Table S1** The leave-one-out sensitivity analysis for identifying heterogeneity.

| Outcome | Heterogeneity source | Sensitivity analysis | | |
| --- | --- | --- | --- | --- |
|  |  | I^2^/% | MD (95% CI) | *P* value |
| ALB | Li L 2021 | 0 | 3.96 (3.47, 4.46) | < 0.00001 |
| TP | Not found | - | - | - |
| TRF | Wan L 2021-1 | 0 | 0.88 (0.77, 0.98) | < 0.00001 |
| GCS | Yang JY 2018 | 0 | 1.55 (1.20, 1.90) | < 0.00001 |
| NIHSS | Not found | - | - | - |
| DAO | Li WW 2024 | 0 | -0.79 (-0.88, -0.70) | < 0.00001 |
| D-LA | Zhang Y 2017 | 0 | -0.09 (-0.10, -0.08) | < 0.00001 |

**Supplementary Table S2** The subgroup analysis based on the preparation.

| Outcome | Subgroup | Number of studies | I^2^/% | MD (95% CI) | *P* value |
| --- | --- | --- | --- | --- | --- |
| ALB | Triple preparation | 5 | 66 | 5.16 (3.54, 6.78) | < 0.00001 |
|  | Quadruple preparation | 4 | 35 | 3.93 (3.08, 4.78) | < 0.00001 |
| TP | Triple preparation | 3 | 87 | 7.84 (3.93, 11.75) | < 0.0001 |
|  | Quadruple preparation | 4 | 98 | 7.14 (1.20, 13.08) | 0.02 |
| TRF | Triple preparation | 1 | 0 | 0.24 (0.01, 0.47) | 0.04 |
|  | Quadruple preparation | 2 | 0 | 0.88 (0.77, 0.98) | < 0.00001 |
| GCS | Triple preparation | 2 | 84 | 2.09 (0.49, 3.49) | 0.003 |
|  | Quadruple preparation | 1 | 0 | 1.60 (1.19, 2.01) | < 0.00001 |
| NIHSS | Triple preparation | 1 | 0 | -2.15 (-3.04, -1.26) | < 0.00001 |
|  | Quadruple preparation | 2 | 95 | -2.18 (-3.97, -0.38) | 0.02 |
| DAO | Triple preparation | - | - | - | - |
|  | Quadruple preparation | 3 | 79 | -0.69 (-0.87, -0.50) | < 0.00001 |
| D-LA | Triple preparation | - | - | - | - |
|  | Quadruple preparation | 3 | 81 | -0.09 (-0.11, -0.08) | < 0.00001 |

**Supplementary Table S3** The subgroup analysis based on the dosages.

| Outcome | Subgroup | Number of studies | I^2^/% | MD (95% CI) | *P* value |
| --- | --- | --- | --- | --- | --- |
| ALB | 0.63 g tid | 4 | 74 | 5.20 (3.44, 6.97) | < 0.00001 |
|  | 1.5 g tid | 4 | 35 | 3.93 (3.08, 4.78) | < 0.00001 |
|  | 2.0 g tid | 1 | 0 | 4.50 (-1.79, 10.79) | 0.16 |
| TP | 0.63 g tid | 3 | 87 | 7.84 (3.93, 11.75) | < 0.0001 |
|  | 1.5 g tid | 4 | 98 | 7.14 (1.20, 13.08) | 0.02 |
|  | 2.0 g tid | - | - | - | - |
| TRF | 0.63 g tid | - | - | - | - |
|  | 1.5 g tid | 2 | 0 | 0.88 (0.77, 0.98) | < 0.00001 |
|  | 2.0 g tid | 1 | 0 | 0.24 (0.01, 0.47) | 0.04 |
| GCS | 0.63 g tid | - | - | - | - |
|  | 1.5 g tid | 1 | 0 | 1.60 (1.19, 2.01) | < 0.00001 |
|  | 2.0 g tid | 2 | 84 | 2.09 (0.69, 3.49) | 0.003 |
| NIHSS | 0.63 g tid | - | - | - | - |
|  | 1.5 g tid | 2 | 95 | -2.18 (-3.97, -0.38) | 0.02 |
|  | 2.0 g tid | 1 | 0 | -2.15 (-3.04, -1.26) | < 0.00001 |
| DAO | 0.63 g tid | - | - | - | - |
|  | 1.5 g tid | 3 | 79 | -0.69 (-0.87, -0.50) | < 0.00001 |
|  | 2.0 g tid | - | - | - | - |
| D-LA | 0.63 g tid | - | - | - | - |
|  | 1.5 g tid | 3 | 81 | -0.09 (-0.11, -0.08) | < 0.00001 |
|  | 2.0 g tid | - | - | - | - |

**Supplementary Table S4** The subgroup analysis based on the treatment duration.

| Outcome | Subgroup | Number of studies | I^2^/% | MD (95% CI) | *P* value |
| --- | --- | --- | --- | --- | --- |
| ALB | 2 weeks | 4 | 0 | 5.00 (3.85, 6.15) | < 0.00001 |
|  | 4 weeks | 5 | 78 | 4.40 (3.14, 5.67) | < 0.00001 |
| TP | 2 weeks | 3 | 97 | 8.41 (0.02, 16.81) | 0.05 |
|  | 4 weeks | 4 | 94 | 6.59 (3.05, 10.12) | 0.0003 |
| TRF | 2 weeks | 2 | 95 | 0.56 (-0.06, 1.18) | 0.08 |
|  | 4 weeks | 1 | 0 | 0.88 (0.74, 1.02) | < 0.00001 |
| GCS | 2 weeks | - | - | - | - |
|  | 4 weeks | 3 | 71 | 1.86 (1.17, 2.56) | < 0.00001 |
| NIHSS | 2 weeks | 1 | 0 | -2.15 (-3.04, -1.26) | < 0.00001 |
|  | 4 weeks | 2 | 95 | -2.18 (-3.97, -0.38) | 0.02 |
| DAO | 2 weeks | 2 | 0 | -0.79 (-0.88, -0.70) | < 0.00001 |
|  | 4 weeks | 1 | 0 | -0.48 (-0.66, -0.30) | < 0.00001 |
| D-LA | 2 weeks | 2 | 86 | -0.10 (-0.12, -0.08) | < 0.00001 |
|  | 4 weeks | 1 | 0 | -0.08 (-0.10, -0.06) | < 0.00001 |
